# Catestatin and Diabetic Retinopathy in Type 2 Diabetes Mellitus

**DOI:** 10.3390/biomedicines14030648

**Published:** 2026-03-13

**Authors:** Karla Katić, Josip Katić, Marko Kumrić, Joško Božić, Daniela Šupe Domić, Ivan Aranza, Šime Kabić, Ivana Barun, Kajo Bućan

**Affiliations:** 1 Department of Ophthalmology, University Hospital of Split, 21000 Split, Croatia; skabic@kbsplit.hr (Š.K.); ivana.bonacin@gmail.com (I.B.); kajobucan@gmail.com (K.B.); 2 Department of Cardiovascular Diseases, University Hospital of Split, 21000 Split, Croatia; josipkati@gmail.com (J.K.); marko.kumric@mefst.hr (M.K.); aranza.ivan@gmail.com (I.A.); 3 Department of Pathophysiology, University of Split School of Medicine, 21000 Split, Croatia; josko.bozic@mefst.hr; 4 Department of Medical Laboratory Diagnostics, University Hospital of Split, 21000 Split, Croatia; daniela.supedomic@gmail.com; 5 Department of Health Studies, University of Split, 21000 Split, Croatia

**Keywords:** catestatin, type 2 diabetes mellitus, diabetic retinopathy, microvascular complications, arterial hypertension, biomarkers

## Abstract

**Background:** Diabetic retinopathy (DR) is the most common microvascular complication of type 2 diabetes mellitus (T2DM). Catestatin (CST) has been implicated in cardiovascular physiology and autonomic modulation, but its role in diabetic microvascular disease remains unclear. **Methods:** In this cross-sectional study, serum CST levels were compared between healthy controls (*n* = 59) and patients with T2DM (*n* = 91), and further between T2DM patients with (*n* = 63) and without DR (*n* = 28). **Results:** CST levels did not differ between T2DM and healthy controls (16 vs. 14 ng/mL; *p* = 0.693). Among normotensive individuals, healthy controls had the lowest CST levels (6 ng/mL), whereas normotensive T2DM participants showed significantly higher concentrations (11 ng/mL; *p* = 0.004). Participants with DR exhibited significantly higher CST than those without DR (14 vs. 10 ng/mL; *p* = 0.034). In the total cohort, arterial hypertension (AH) was the strongest independent predictor of CST (B = 0.562; *p* < 0.001). In the multivariable model restricted to T2DM patients, DR emerged as the sole independent determinant of CST (B = 0.251; *p* = 0.028), whereas AH was no longer significant (*p* = 0.581). **Conclusions:** Circulating catestatin levels were not associated with the presence of T2DM itself but were significantly elevated in patients with DR. These findings suggest that CST may reflect microvascular and neurovascular stress rather than metabolic status and could represent a biomarker of diabetic microangiopathy.

## 1. Introduction

Type 2 diabetes mellitus (T2DM) is a chronic, progressive metabolic disorder marked by insulin resistance, β-cell dysfunction, and sustained hyperglycemia, which in turn lead to multiple long-term complications [1]. It affects hundreds of millions of individuals worldwide and represents one of the leading causes of morbidity due to its microvascular and macrovascular complications [2,3]. Chronic hyperglycemia induces a cascade of pathological processes including oxidative stress, endothelial dysfunction, chronic low-grade inflammation, impaired nitric oxide bioavailability, and autonomic imbalance that together drive structural and functional damage in target organs [1,4]. Among microvascular complications, diabetic retinopathy (DR) is significant as it remains the most frequent cause of preventable blindness in working-age adults [5,6]. Globally, approximately one-third of individuals with diabetes show signs of diabetic retinopathy, and about one in ten develop vision-threatening disease [6]. Although classical risk factors for DR, such as duration of diabetes, glycemic control, hypertension, and dyslipidemia, are well known, the molecular mediators linking metabolic dysregulation to microvascular injury are incompletely understood [2,3,5]. Emerging evidence indicates that diabetic retinopathy begins with early retinal neurovascular dysfunction and neuronal impairment preceding clinically visible vascular lesions [7]. Therefore, there is growing interest in circulating biomarkers that reflect early endothelial dysfunction, autonomic imbalance, or inflammatory stress, which could help identify diabetic patients at risk for microvascular complications.

One such biomarker is catestatin (CST), a 21-amino-acid peptide derived from the precursor protein chromogranin A (CgA) [8]. CST plays a multifunctional physiological role: it is a potent endogenous inhibitor of catecholamine release, regulator of autonomic cardiovascular function, modulator of immune responses, and mediator of endothelial and angiogenic activity [8]. CST exhibits anti-inflammatory, antioxidative, vasodilatory, and insulin-sensitizing properties [8,9,10]. Reduced CST levels have been reported in metabolic syndrome, obesity, and insulin resistance, suggesting that CST reflects disturbances in metabolic homeostasis and sympathoadrenal activation [9,10]. Recent research has highlighted a potential role of CST in ocular physiology. CST has been shown to exert angiogenic actions via basic fibroblast growth factor–dependent pathways, to modulate retinal neuronal signaling, and to influence vascular remodeling and promote agiogenesis in ischemic tissues [11,12]. Despite increasing experimental evidence regarding the vascular and neuroendocrine actions of CST, clinical data evaluating its circulating levels in diabetic microvascular complications remain limited [13]. It is unclear whether CST alterations are related to the presence of T2DM or to the development of microvascular complications like DR. Moreover, arterial hypertension, a major activator of sympathoadrenal pathways, may substantially influence circulating CST concentrations and confound their interpretation in diabetic populations [14]. Therefore, clinical studies simultaneously evaluating diabetes status, microvascular involvement, and blood pressure are needed. The aim of this study was to compare serum CST levels between individuals with and without T2DM, and further between T2DM patients with and without DR. In addition, we examined these differences specifically in individuals without known arterial hypertension, to better understand the microvascular rather than blood pressure-related determinants of CST serum levels.

## 2. Methods

This was a cross-sectional study with predefined group comparisons, including patients with T2DM with and without DR and age- and sex-matched healthy controls. It was conducted at the Department of Ophthalmology, University Hospital of Split in Croatia from August 2023 to August 2024. The study protocol was approved by the Ethics Committee of the University Hospital Centre Split (Approval No 2181-147/01/06/LJ.Z.-23-06) and conducted in accordance with the principles of the Declaration of Helsinki.

This study included 91 patients with T2DM and 59 age- and sex-matched healthy control subjects. Sample size estimation was performed based on the results of a pilot study that included 10 patients with T2DM and 10 healthy control subjects. Serum catestatin concentration was defined as the primary outcome. By comparing mean catestatin levels between groups, assuming a two-sided type I error rate of 0.05 and a target study power of 80%, the minimal required sample size was estimated to detect a clinically relevant difference between the examined groups. Ultimately, a total of 150 participants were included in the study (59 healthy controls and 91 patients with T2DM), fulfilling the calculated requirements. Patients with T2DM were consecutively recruited from the outpatient ophthalmology clinic during the study period and screened for eligibility according to predefined inclusion and exclusion criteria. Healthy control subjects were also recruited from outpatient ophthalmology clinic, matched to the T2DM group by age and sex, and the same exclusion criteria were applied in their selection as for the study groups. All participants voluntarily signed written informed consent forms and were informed about the aims and procedures of the study before enrollment. All subjects were aged 18 years or older, had a body mass index (BMI) below 35 kg/m^2^, and had been on stable medication regimens for at least three months before inclusion.

Exclusion criteria were acute systemic or ocular infection or inflammation, active cardiovascular disease, chronic kidney or liver disease, malignancy, use of medications known to influence catestatin levels (e.g., corticosteroids), vitreous opacities or visually significant cataracts that impaired fundus examination, proliferative diabetic retinopathy, previous vitreoretinal surgery or laser photocoagulation, and ocular surgery within the previous six months.

Patients with T2DM were diagnosed according to the American Diabetes Association (ADA) criteria [15]. The diagnosis was established if at least one of the following criteria was met: glycated hemoglobin (HbA1c) ≥ 6.5%, fasting plasma glucose ≥ 7.0 mmol/L, or current use of oral hypoglycemic agents and/or insulin therapy regardless of current laboratory values.

Arterial hypertension (AH) was defined according to the criteria outlined in the ESC/ESH guidelines as systolic blood pressure ≥ 140 mmHg and/or diastolic blood pressure ≥ 90 mmHg measured at rest [16]. Additionally, all participants receiving antihypertensive therapy were classified as hypertensive irrespective of current blood pressure values.

All participants underwent a detailed medical interview and physical examination. Body weight, height, and body mass index (BMI) were measured using a calibrated medical scale with a built-in stadiometer (Seca, Birmingham, UK), and BMI was calculated as weight (kg) divided by height squared (m^2^).

Blood pressure was measured using a standard Riester Big Ben Aneroid sphygmomanometer (Rudolf Riester GmbH, Jungingen, Germany). Blood pressure measurements were obtained after participants had rested in a seated position for at least 10 min. The mean value of two consecutive measurements was used for analysis. Medical records were reviewed to extract relevant clinical data.

Each participant also underwent a complete ophthalmologic evaluation including best-corrected visual acuity (BCVA) using a Snellen chart, fundus examination, and macular optical coherence tomography (OCT). OCT imaging was performed with the CIRRUS^®^ 6000 OCT system (Carl Zeiss, Oberkochen, Germany). In the first group of patients with T2DM there were no clinical signs of diabetic retinopathy on ophthalmologic examination. In individuals with DR, the severity of DR was graded according to the International Clinical Diabetic Retinopathy and Diabetic Macular Edema Disease Severity Scale [17].

Venous blood samples were drawn in the morning after an overnight fasting period of 8–12 h. Serum was separated by centrifugation and stored at −80 °C until analysis. Circulating catestatin concentrations were measured using a commercial ELISA assay (EK-053-27CE, EIA kit, Phoenix pharmaceuticals Inc., Burlingame, CA, USA, SAD) according to the manufacturer’s instructions on an automated biochemical analyzer (Beckman Coulter Inc., Miami, FL, USA) at the Department of Medical Laboratory Diagnostics, University Hospital of Split. According to the technical specifications of the assay, the sensitivity for catestatin was 0.05 ng/mL, with a linear detection range of 0.05–0.92 ng/mL and an overall measuring range of 0–100 ng/mL. The intra-assay coefficient of variation was <10%, while the total assay variability was <15%. The laboratory personnel were blinded to participants’ clinical group assignments. Routine biochemical parameters were determined using standard automated laboratory methods.

## 3. Statistical Analysis

Statistical analyses were conducted using SPSS software, version 20 (IBM Corp.). A statistically significant departure from a normal (Gaussian) distribution was identified for most variables according to the Shapiro–Wilk test. Consequently, all numerical variables are reported as medians with corresponding interquartile ranges (IQRs). Categorical variables are presented as absolute frequencies with their proportional representation in the total sample. Comparisons of numerical variables were performed using the Mann–Whitney U test (for two groups) and the Kruskal–Wallis test with Dunn’s post hoc procedure for multiple comparisons, with *p*-values adjusted using the Bonferroni correction (for analyses involving more than two groups). Multiple linear regression analysis was applied to determine which variables independently predicted catestatin levels. Results are expressed as unstandardized regression coefficients (B) with 95% confidence intervals. All statistical tests were two-tailed, and statistical significance was defined as *p* < 0.05.

## 4. Results

This study included 150 patients in total, 91 patients with T2DM (28 patients without any signs of DR, and 63 patients with NPDR), and 59 healthy, age- and sex-matched controls. The baseline characteristics of patients are shown in Table 1.

When comparing serum catestatin concentrations between individuals with T2DM and healthy control subjects, no statistically significant difference was observed. Healthy controls demonstrated a median CST level of 16 ng/mL (IQR 8–23), whereas participants with diabetes had a median value of 14 ng/mL (IQR 8–26) (*p* = 0.693) (Table 2).

Patients with T2DM but without DR had a median CST level of 10 ng/mL (IQR 7–25), whereas those with DR showed significantly higher values of 14 ng/mL (IQR 10–26; *p* = 0.034) (Table 2).

Healthy controls without AH exhibited the lowest CST levels of 6 ng/mL (IQR 3–8), while individuals with T2DM without AH had significantly higher concentrations of 11 ng/mL (IQR 7–19; *p* = 0.004).

In the multivariable linear regression model applied to the entire study population, AH was identified as the strongest independent predictor of serum catestatin concentrations. After adjusting for diabetes status, sex, age, and BMI, AH was associated with a markedly higher CST level (B = 0.562, *p* < 0.001), corresponding to approximately a threefold increase relative to normotensive individuals (Table 3). T2DM was also an independent predictor of higher CST (B = 0.385, *p* < 0.001), although its effect size was smaller than that of AH. Importantly, the interaction term T2DM × AH was statistically significant and negative (B = −0.512, *p* < 0.001), demonstrating that the AH effect on CST is substantially attenuated within the T2DM subgroup. In other words, while AH strongly elevates catestatin in the general population, this effect is diminished in individuals with T2DM, indicating that the combined effects of T2DM and AH are not additive. This finding is consistent with descriptive analyses showing disproportionately high CST levels in hypertensive non-diabetic subjects. The model showed good explanatory capacity (adjusted R^2^ = 0.301), and no substantial collinearity was detected among predictors.

In the multivariable linear regression model performed exclusively within the T2DM cohort, the predictors included AH, sex, age, BMI, HbA1c, LDL cholesterol, estimated glomerular filtration rate (eGFR), and the presence of DR. After full adjustment, DR emerged as the only independent predictor of serum catestatin levels (B = 0.251, *p* = 0.028) (Table 3).

CST levels did not reach a statistically significant difference across the stages of NPDR (*p* = 0.106), although an increase was observed in more severe stages.

## 5. Discussion

In this study, we demonstrated that serum CST levels did not differ significantly between T2DM patients and healthy controls, indicating that diabetes itself is not associated with altered CST concentrations. In the multivariable regression model performed in the total study population, AH emerged as the strongest independent predictor of higher CST levels, while its effect was markedly attenuated in the presence of T2DM, indicating a non-additive interaction. Within the separate multivariable model restricted to the T2DM cohort, only DR remained independently associated with CST levels, while CST showed a non-significant upward trend across increasing NPDR severity.

Long-standing hyperglycemia is known to impair sympathetic–adrenal signaling, modify chromogranin A processing, and induce autonomic neuropathy, all of which may blunt catecholamine-dependent CST secretion [8,9,18]. In addition, T2DM and AH share overlapping biological pathways—including oxidative stress, chronic low-grade inflammation, reduced nitric oxide bioavailability, and microvascular remodeling [13,14,19]. These mechanisms may partly explain the associations observed in our analysis. As a consequence, the incremental CST rise typically observed in hypertensive non-diabetic individuals may be diminished in the diabetic milieu, explaining why CST did not differ between T2DM and controls in the overall cohort. Taken together, these findings indicate that CST regulation may shift from reflecting systemic hemodynamic stress in the general population to reflecting microvascular and neurovascular dysfunction once diabetes and its complications develop.

The analysis of normotensive subgroups further reinforces this interpretation. Healthy controls without AH displayed the lowest CST values in the entire sample, representing a physiological baseline with minimal vascular or metabolic stress. Even within this restricted comparison, normotensive T2DM had markedly higher CST concentrations, suggesting that subtle neurohumoral and microvascular changes associated with T2DM may stimulate CST secretion. This gradient supports the hypothesis that CST elevation reflects cumulative microvascular or neurohumoral stress, rather than metabolic status alone, and that removal of AH as a confounder strengthens the association between CST and T2DM-related vascular injury.

A markedly different pattern emerged when microvascular status was examined. In our study, participants with DR exhibited significantly higher CST concentrations than T2DM participants without DR. The multivariable regression exclusively within the T2DM cohort demonstrated that DR was the only independent predictor of serum CST, remaining statistically significant. AH, which was the dominant determinant in the entire population, lost its predictive value within the T2DM subgroup, indicating that the regulatory influence of CST shifts from hemodynamic to microvascular domains once T2DM is established. The absence of a significant interaction between DR and AH further indicates that AH does not alter or amplify the effect of DR on CST levels. Diabetic retinopathy is characterized by retinal capillary non-perfusion and tissue hypoxia, which activate neurovascular and angiogenic signaling pathways [7]. Experimental studies have shown that catestatin acts as an angiogenic peptide through a basic fibroblast growth factor–dependent mechanism and is expressed in retinal neuronal tissue [11,12]. Elevated circulating CST likely reflects a systemic response to local retinal ischemia and neurovascular unit dysfunction. Our results support earlier reports indicating that CST has anti-inflammatory and endothelium-modulating properties and acts as a mediator of vascular injury and microvascular stress [8,20,21]. The angiogenic activity of CST is particularly relevant because retinal ischemia in DR precedes overt neovascularization, suggesting that circulating neurovascular peptides may increase during early stages of retinal injury [7].

In non-diabetic individuals, AH is associated with increased sympathoadrenal activation and catecholamine release, which stimulates chromogranin A processing and CST secretion as part of a negative feedback mechanism [19]. However, long-standing T2DM is frequently accompanied by autonomic neuropathy and impaired neuroendocrine responsiveness [20,21]. Consequently, the physiological relationship between blood pressure and CST may become attenuated.

Data regarding CST in other diabetic microvascular complications remain scarce. However, given its links with vascular dysfunction and autonomic neuropathy, CST alterations may not be limited to retinal disease alone [22,23]. Similar neurovascular and microvascular injury mechanisms are present in diabetic neuropathy and nephropathy [22,23], suggesting that CST may reflect broader microvascular stress in diabetes, although dedicated studies are required.

From a clinical perspective, if confirmed in prospective studies, CST measurement could contribute to risk stratification by identifying diabetic patients at increased risk of developing retinopathy or may assist in monitoring progression of microvascular disease. However, CST concentrations are also influenced by systemic cardiovascular conditions, including hypertension and coronary artery disease. Therefore, its clinical value would most likely depend on interpretation within a defined clinical context and in combination with ophthalmologic assessment rather than as a stand-alone laboratory test.

This study has several strengths. The cohort was clinically well characterized, and the presence of diabetic retinopathy was confirmed by detailed ophthalmologic examination and optical coherence tomography. We included both diabetic and non-diabetic participants and performed multivariable analyses adjusting for major confounders. Additionally, analysis of normotensive subgroups allowed better separation of microvascular effects from blood pressure–related influences.

Certain limitations of this study need to be considered. First, it was conducted as a single-center study and included a relatively small sample size. Second, the study population consisted exclusively of Caucasian participants, which may restrict applicability of our findings to other ethnic groups. Third, due to the cross-sectional design, causal relationships between catestatin levels and diabetic retinopathy cannot be established. Finally, larger multicenter and longitudinal studies are needed to confirm these findings.

To the best of our knowledge, this is the first investigation to examine catestatin levels in diabetic individuals with and without diabetic retinopathy, as well as in healthy subjects.

## 6. Conclusions

Circulating catestatin concentrations were not associated with the presence of T2DM itself but were significantly elevated in patients with DR. Within the diabetic population, retinopathy rather than AH emerged as the main determinant of CST levels. Its elevation in DR may reflect a compensatory vasoprotective response to retinal neurovascular dysfunction. Accordingly, circulating CST may represent a potential indicator of diabetic microvascular involvement. Future longitudinal studies are required to determine whether CST has prognostic value for early detection, progression risk stratification, or therapeutic monitoring in DR.

## Figures and Tables

**Table 1:** Demographics and baseline clinical parameters.

**All Participants**			
**Variables**	**Without Diabetes Mellitus Type 2** **(*N* = 59)**	**With Diabetes** **Mellitus Type 2** **(*N* = 91)**	***p* Value**
**Sex, male**	33 (56)	56 (50)	0.521
**Age, years**	65 (60–70)	69 (61–74)	0.008
**Body mass index**	27 (25–30)	28 (26–30)	0.284
**Arterial hypertension**	38 (64)	85 (79)	0.152
**Participants with diabetes mellitus type 2**			
	**Without diabetic retinopathy** **(*N* = 28)**	**With diabetic retinopathy** **(*N* = 63)**	
**Sex, male**	24 (85)	32 (46)	0.437
**Age, years**	71 (65–77)	68 (59–73)	0.047
**Body mass index**	29 (27–31)	27 (25–30)	0.025
**Arterial hypertension**	34 (79)	51 (74)	0.651
**Duration of diabetes, years**	10 (5–18)	17 (10–25)	0.002
**HbA1c**	7(6–8)	7 (7–8)	0.172
**eGFR**	82 (70–93)	75.1 (56–86)	0.030
**Visual acuity RE (Snellen)**	0.9 (0.5–1.0.9)	0.4 (0.1–0.5)	<0.001
**Visual acuity LE (Snellen)**	0.8 (0.4–0.9)	0.4 (0.3–0.5)	<0.001
**CMT RE**	259 (242–283)	413 (351–486)	<0.001
**CMT LE**	259 (239–280)	439 (370–479)	<0.001
**MV RE**	9.9 (9.4–10.4)	11 (10–12.6)	<0.001
**MV LE**	9.9 (9.6–10.3)	11.8 (10.4–12.6)	<0.001

Data presented as median (IQR) or N (%). HbA1c—glycated hemoglobin; eGFR—estimated glomerular filtration rate; RE—right eye; LE—left eye; CMT—central macular thickness; MV—macular volume.

**Table 2:** Comparison of serum catestatin levels between groups.

All Participants				Participants Without Diabetes Mellitus Type 2			Participants with Diabetes Mellitus Type 2		
	Without Diabetes Mellitus Type 2 (*N* = 59)	With Diabetes Mellitus Type 2 (*N* = 91)	*p* Value	No Arterial Hypertension (*N* = 21)	Arterial Hypertension (*N* = 38)	*p* Value	Without Diabetic Retinopathy (*N* = 28)	With Diabetic Retinopathy (*N* = 63)	*p* Value
**CST**	16 (8–23)	14 (8–26)	0.693	6 (3–8)	20 (16–28)	<0.001	10 (7–25)	14 (10–26)	0.034

Data presented as median (IQR).

**Table 3:** Multivariable regression analysis.

Variable	Standardized β	*p* Value
**All participants**		
**Diabetes** **mellitus type 2**	0.385	**<0.001**
**Arterial hypertension**	0.562	**<0.001**
**Sex**	−0.094	0.085
**Age**	0.007	0.080
**Body mass index**	0.008	0.185
**DM × AT interaction**	−0.512	**<0.001**
**Participants with diabetes** **mellitus type 2**		
**Arterial hypertension**	0.060	0.581
**Sex**	−0.085	0.448
**Age**	0.148	0.249
**Body mass index**	0.200	0.069
**Diabetic retinopathy**	0.251	**0.028**
**HbA1c**	0.062	0.558
**LDL cholesterol**	−0.162	0.118
**eGFR**	−0.183	0.122

DM × AT—diabetes mellitus and arterial hypertension; HbA1c—glycated hemoglobin; LDL cholesterol—low-density lipoprotein cholesterol; eGFR—estimated glomerular filtration rate. Bold *p* values denote statistical significance.

## Data Availability

The data presented in this study are available upon request to the corresponding author. The data are not publicly available because some of the data sets will be used for further research.

## References

[1] Domingueti C.P., Dusse L.M., Carvalho M.D., de Sousa L.P., Gomes K.B., Fernandes A.P. (2016). Diabetes mellitus: The linkage between oxidative stress, inflammation, hypercoagulability and vascular complications. J. Diabetes Complicat..

[2] Sun H., Saeedi P., Karuranga S., Pinkepank M., Ogurtsova K., Duncan B.B., Stein C., Basit A., Chan J.C.N., Mbanya J.C. (2022). IDF Diabetes Atlas: Global, regional and country-level diabetes prevalence estimates for 2021 and projections for 2045. Diabetes Res. Clin. Pract..

[3] Młynarska E., Czarnik W., Dzieża N., Jędraszak W., Majchrowicz G., Prusinowski F., Stabrawa M., Rysz J., Franczyk B. (2025). Type 2 Diabetes Mellitus: New Pathogenetic Mechanisms, Treatment and the Most Important Complications. Int. J. Mol. Sci..

[4] Weinberg Sibony R., Segev O., Dor S., Raz I. (2024). Overview of oxidative stress and inflammation in diabetes. J. Diabetes.

[5] Li H., Liu X., Zhong H., Fang J., Li X., Shi R., Yu Q. (2023). Research progress on the pathogenesis of diabetic retinopathy. BMC Ophthalmol..

[6] Teo Z.L., Tham Y.C., Yu M., Chee M.L., Rim T.H., Cheung N., Bikbov M.M., Wang Y.X., Tang Y., Lu Y. (2021). Global Prevalence of Diabetic Retinopathy and Projection of Burden through 2045: Systematic Review and Meta-analysis. Ophthalmology.

[7] Simó R., Hernández C., European Consortium for the Early Treatment of Diabetic Retinopathy (EUROCONDOR) (2014). Neurodegeneration in the diabetic eye: New insights and therapeutic perspectives. Trends Endocrinol. Metab..

[8] Bourebaba Y., Mularczyk M., Marycz K., Bourebaba L. (2021). Catestatin peptide of chromogranin A as a potential new target for several risk factors management in the course of metabolic syndrome. Biomed. Pharmacother..

[9] Simunovic M., Supe-Domic D., Karin Z., Degoricija M., Paradzik M., Bozic J., Unic I., Skrabic V. (2019). Serum catestatin concentrations are decreased in obese children and adolescents. Pediatr. Diabetes.

[10] Ying W., Mahata S., Bandyopadhyay G.K., Zhou Z., Wollam J., Vu J., Mayoral R., Chi N.W., Webster N.J.G., Corti A. (2018). Catestatin Inhibits Obesity-Induced Macrophage Infiltration and Inflammation in the Liver and Suppresses Hepatic Glucose Production, Leading to Improved Insulin Sensitivity. Diabetes.

[11] Theurl M., Schgoer W., Albrecht K., Jeschke J., Egger M., Beer A.G., Vasiljevic D., Rong S., Wolf A.M., Bahlmann F.H. (2010). The neuropeptide catestatin acts as a novel angiogenic cytokine via a basic fibroblast growth factor-dependent mechanism. Circ. Res..

[12] Gramlich O.W., Lorenz K., Grus F.H., Kriechbaum M., Ehrlich D., Humpel C., Fischer-Colbrie R., Bechrakis N.E., Troger J. (2014). Catestatin-like immunoreactivity in the rat eye. Neuropeptides.

[13] Kulpa J., Paduch J., Szczepanik M., Gorący-Rosik A., Rosik J., Tchórz M., Pawlik A., Gorący J. (2025). Catestatin in Cardiovascular Diseases. Int. J. Mol. Sci..

[14] Bozic J., Kumric M., Ticinovic Kurir T., Urlic H., Martinovic D., Vilovic M., Tomasovic Mrcela N., Borovac J.A. (2021). Catestatin as a Biomarker of Cardiovascular Diseases: A Clinical Perspective. Biomedicines.

[15] American Diabetes Association Professional Practice Committee (2025). 2. Diagnosis and Classification of Diabetes: Standards of Care in Diabetes-2025. Diabetes Care.

[16] McEvoy J.W., McCarthy C.P., Bruno R.M., Brouwers S., Canavan M.D., Ceconi C., Christodorescu R.M., Daskalopoulou S.S., Ferro C.J., Gerdts E. (2024). 2024 ESC Guidelines for the management of elevated blood pressure and hypertension. Eur. Heart J..

[17] Wilkinson C.P., Ferris F.L., Klein R.E., Lee P.P., Agardh C.D., Davis M., Dills D., Kampik A., Pararajasegaram R., Verdaguer J.T. (2003). Global Diabetic Retinopathy Project Group. Proposed international clinical diabetic retinopathy and diabetic macular edema disease severity scales. Ophthalmology.

[18] Vinik A.I., Ziegler D. (2007). Diabetic cardiovascular autonomic neuropathy. Circulation.

[19] Mahata S.K., Mahata M., Fung M.M., O’Connor D.T. (2010). Catestatin: A multifunctional peptide from chromogranin A. Regul. Pept..

[20] Muntjewerff E.M., Epremidze D., Nezhyva M., Kal S., Rohm T.V., Tang K., Singh K., Espes D., Jati S., Bootsma M. (2025). Chromogranin A and catestatin regulate pancreatic islet homeostasis, endocrine function, and neurotransmitter signaling. Commun. Biol..

[21] Dasgupta A., Bandyopadhyay G.K., Ray I., Bandyopadhyay K., Chowdhury N., De R.K., Mahata S.K. (2020). Catestatin improves insulin sensitivity by attenuating endoplasmic reticulum stress: In vivo and in silico validation. Comput. Struct. Biotechnol. J..

[22] Watanabe T. (2021). The Emerging Roles of Chromogranins and Derived Polypeptides in Atherosclerosis, Diabetes, and Coronary Heart Disease. Int. J. Mol. Sci..

[23] Spallone V. (2019). Update on the Impact, Diagnosis and Management of Cardiovascular Autonomic Neuropathy in Diabetes: What Is Defined, What Is New, and What Is Unmet. Diabetes Metab. J..

